# Drug resistant tuberculosis: Implications for transmission, diagnosis, and disease management

**DOI:** 10.3389/fcimb.2022.943545

**Published:** 2022-09-23

**Authors:** Dale Liebenberg, Bhavna Gowan Gordhan, Bavesh Davandra Kana

**Affiliations:** Department of Science and Innovation/National Research Foundation (DSI/NRF) Centre of Excellence for Biomedical Tuberculosis Research (TB), Faculty of Health Sciences, University of the Witwatersrand, National Health Laboratory Service, Johannesburg, South Africa

**Keywords:** acquired drug resistance, transmitted drug resistance, health systems strengthening, persisters, tolerance

## Abstract

Drug resistant tuberculosis contributes significantly to the global burden of antimicrobial resistance, often consuming a large proportion of the healthcare budget and associated resources in many endemic countries. The rapid emergence of resistance to newer tuberculosis therapies signals the need to ensure appropriate antibiotic stewardship, together with a concerted drive to develop new regimens that are active against currently circulating drug resistant strains. Herein, we highlight that the current burden of drug resistant tuberculosis is driven by a combination of ongoing transmission and the intra-patient evolution of resistance through several mechanisms. Global control of tuberculosis will require interventions that effectively address these and related aspects. Interrupting tuberculosis transmission is dependent on the availability of novel rapid diagnostics which provide accurate results, as near-patient as is possible, together with appropriate linkage to care. Contact tracing, longitudinal follow-up for symptoms and active mapping of social contacts are essential elements to curb further community-wide spread of drug resistant strains. Appropriate prophylaxis for contacts of drug resistant index cases is imperative to limit disease progression and subsequent transmission. Preventing the evolution of drug resistant strains will require the development of shorter regimens that rapidly eliminate all populations of mycobacteria, whilst concurrently limiting bacterial metabolic processes that drive drug tolerance, mutagenesis and the ultimate emergence of resistance. Drug discovery programs that specifically target bacterial genetic determinants associated with these processes will be paramount to tuberculosis eradication. In addition, the development of appropriate clinical endpoints that quantify drug tolerant organisms in sputum, such as differentially culturable/detectable tubercle bacteria is necessary to accurately assess the potential of new therapies to effectively shorten treatment duration. When combined, this holistic approach to addressing the critical problems associated with drug resistance will support delivery of quality care to patients suffering from tuberculosis and bolster efforts to eradicate this disease.

## Introduction

The growing spread of antimicrobial resistance (AMR) will most certainly undermine delivery of effective healthcare globally, necessitating a multipronged approach that accelerates development of new antimicrobials, together with careful deployment of these life-saving treatments in a more regulated manner (Nathan, 2020). Effective stewardship of antibiotics in plants and animals through a One-Health approach is also central to future efforts for combating AMR. Whilst antibiotic resistant bacteria and other pathogens comprise a notable proportion of resistance, drug resistant tuberculosis (TB) accounts for a disproportionately large amount of the global AMR burden. Much of this comprises circulation of *Mycobacterium tuberculosis* strains resistant to rifampicin and isoniazid (commonly referred to as multidrug resistance, MDR, **Box 1**) or strains that are MDR with added resistance to second line agents including fluoroquinolones and aminoglycosides (extensively drug resistant, XDR-TB, **Box 1**). Given that many health systems have opted to discontinue use of aminoglycosides, this definition of XDR tuberculosis will soon be outdated and possibly replaced by one that refers to the WHO drug category and resistance pattern (Dheda et al., 2019). Currently available agents for drug resistant TB, classified into groups A-C are shown in **Box 1**. Monoresistance to rifampicin or isoniazid is also a growing problem in TB endemic regions (Variava and Martinson, 2018). In 2020, 71% (2.1 of 3.0 million) of people diagnosed with bacteriologically confirmed pulmonary TB were also tested for rifampicin resistance (RR). Among these, 132 222 cases of MDR/RR-TB and 25 681 cases of pre-XDR-TB (MDR TB plus resistance to any fluoroquinolone or an injectable, **Box 1**) or XDR-TB were reported (World Health Organization, 2021a). In addition to drug resistance, HIV co-infection is another primary driver of poor TB outcomes with 214 000 deaths occurring among people living with HIV (PLWHIV), in addition to the 1.3 million worldwide TB deaths in 2020 (World Health Organization, 2021a). Moreover, any gains that global TB programs made during the last decade have been severely undermined by Covid-19, the most obvious being an 18% drop (7.1 million to 5.8 million) globally in the number of people newly diagnosed with TB in 2020, compared with 2019 (World Health Organization, 2021a). While the mandated wearing of masks and widespread lockdowns to limit the spread of SARS-CoV-2 might have helped decrease the tuberculosis burden, there are multiple Covid-19-derived factors that have had a negative impact on TB control. These include delayed treatment due to reduced access to public transport and health care facilities, misdiagnosis given symptom similarities between TB and Covid-19, disruptions of medical stocks and laboratory services, and the desire to avoid the stigma of disease (Dheda et al., 2022). During the same time, the number of people provided with treatment for drug-resistant TB reduced by 15%, from 177 100 to 150 359, about 1 in 3 of those in need (World Health Organization, 2021a).

Box 1 **Definitions, WHO MDR-TB drug categorization and new TB drug development pipeline.**

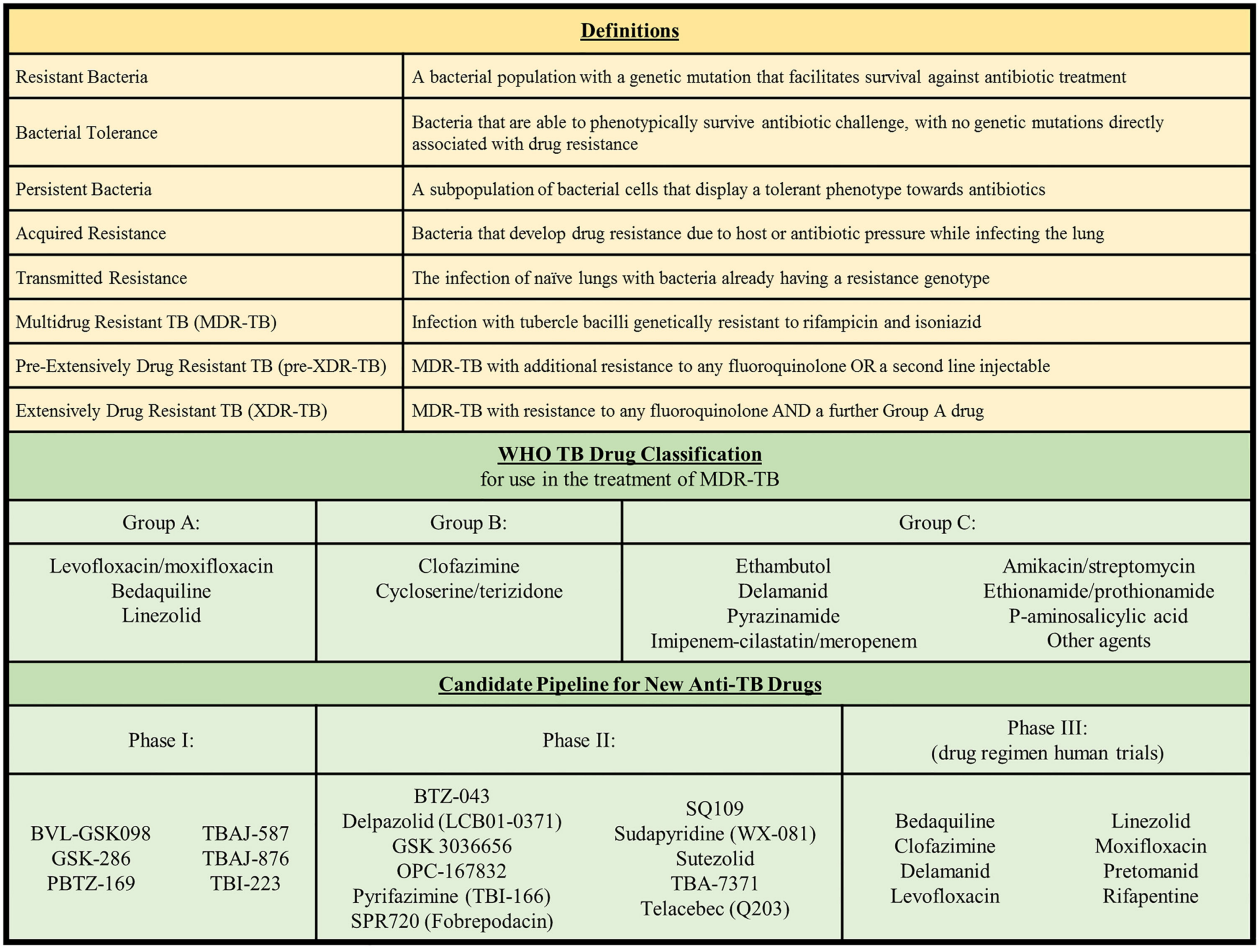

## Transmitted and acquired resistance

**Figure 1:**
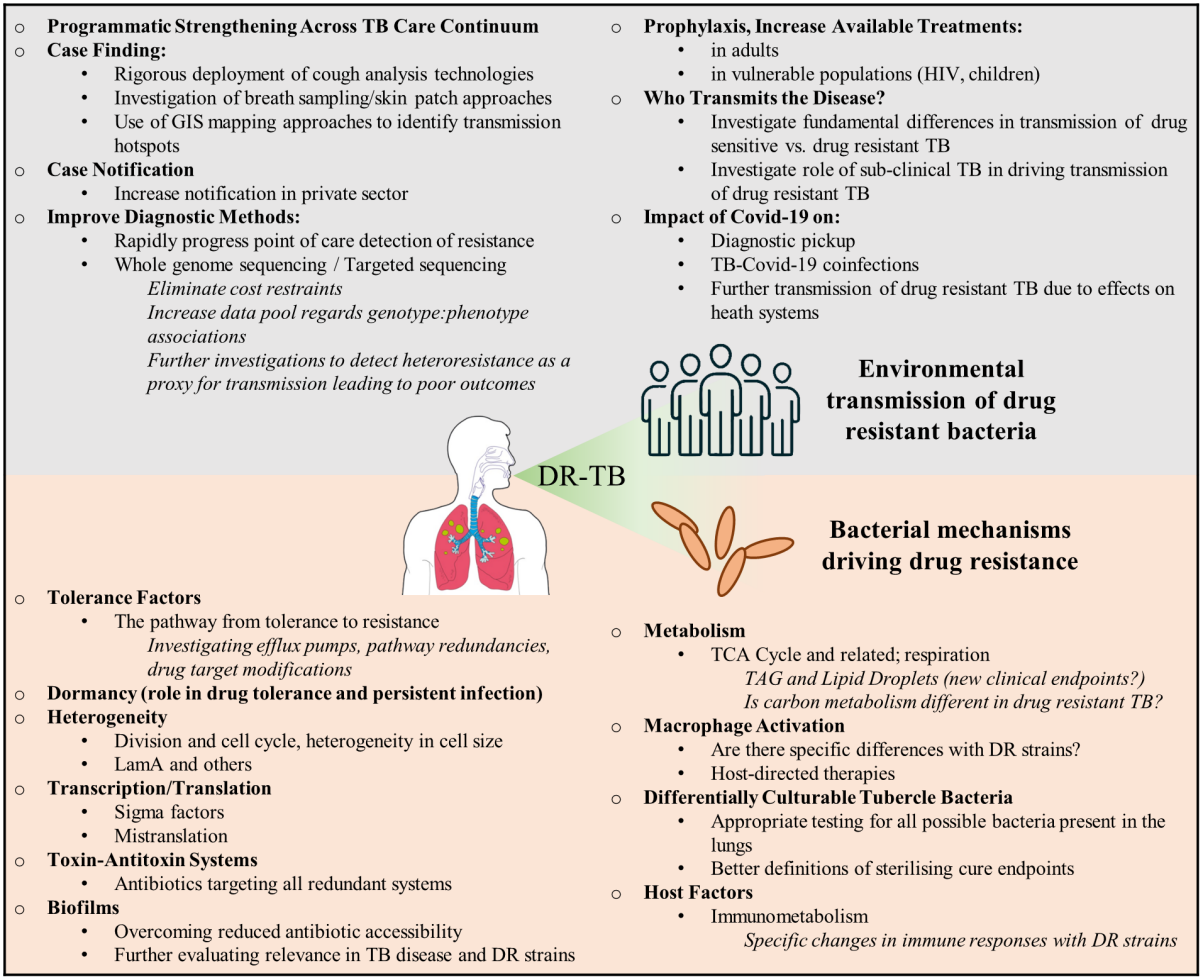
Drivers of drug resistance in TB. Key issues that need to be addressed in the fight against transmitted and acquired drug resistance. These primarily relate to strengthening health care systems to understanding how drug resistance develops.

**Figure 2:**
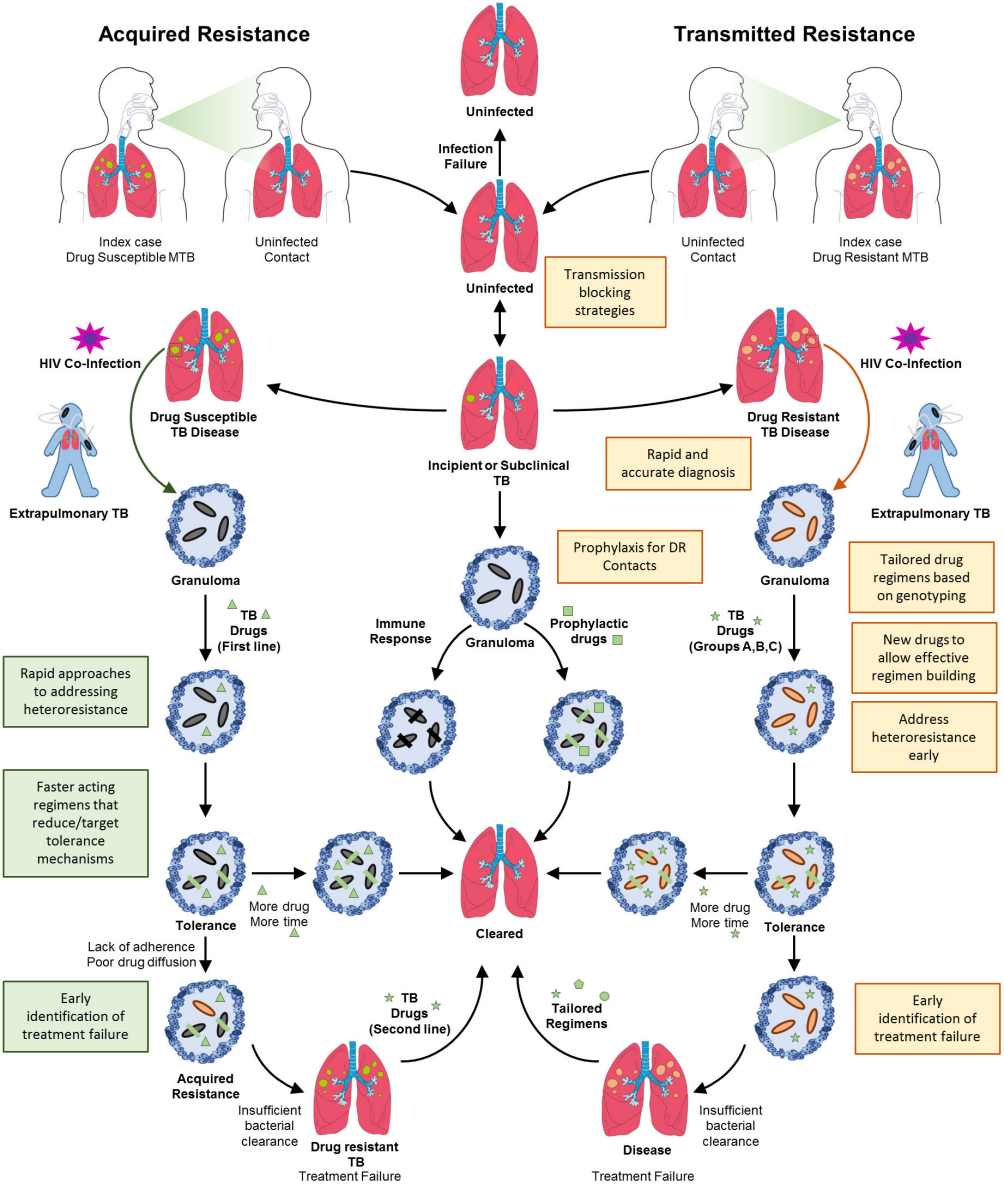
Transmitted and acquired drug resistant TB. Drug resistant TB can arise by two mechanisms, acquired resistance or directly through transmitted resistance. Shown is the natural progression of TB infection and disease, initiated by transmission of bacteria, followed by the development of incipient or subclinical TB. If identified, incipient or subclinical TB can be resolved by administration of prophylaxis or spontaneously. A small proportion of these cases will progress to full blown TB disease in the lungs wherein granulomas necrotize and rupture. Concomitant HIV infection will also result in extrapulmonary TB. In the case of infection with drug sensitive TB, the bacterial population will expand and drug treatment will result in the survival of tolerant organisms that will eventually be cleared with protracted treatment. The phenomenon of drug tolerance highlights the need for additional and faster acting drug treatments. Lack of adherence or poor drug diffusion will result in these tolerant organisms eventually acquiring stable drug resistance, resulting in treatment failure and drug resistant TB. This will necessitate administration of an alternate regimen directed at eliminating resistant organisms. Transmitted resistance occurs when an individual is infected with organisms that are genetically drug resistant. In the absence of diagnostic testing capable of identifying drug resistance mutations for most drugs except rifampicin, the treatment of these patients with first-line drugs will unfortunately result in failure. If identified, together with appropriate susceptibility testing, treatment of drug resistant TB follows a similar sequence of events to that for drug susceptible TB. Given the longer durations of treatment, the chances for evolution of further resistance are high. Hence, the requirement for the correct choice of regimens and development of novel drugs to eliminate these resistant organisms. Shaded boxes indicate the need for new interventions or health system strengthening.

For more than 10 years, estimates of the proportion of people diagnosed for the first time with MDR/RR-TB has remained at about 3–4% and for those previously treated for TB has stayed at about 18–21% (World Health Organization, 2021a). This indicates that a substantive burden of drug resistant TB is driven by on-going transmission. Given this, the quest to eradicate drug resistant TB needs bolstering using approaches that address primary drivers including (I) deployment of transmission blocking strategies, together with novel approaches to case finding, contact tracing and related public health measures and (II) fast-tracking development of new therapeutics with novel modes of action that shorten the duration of treatment, limit emergence of resistance and are active on contemporaneous resistant strains, **Figure 1**. Drug resistant TB can arise either through direct transmission of genetically resistant bacteria (transmitted resistance, **Figure 2**) or intra-patient evolution of resistance (acquired resistance, **Figure 2**). Consequently, strategies to eliminate TB need to address health systems strengthening in a manner that limits community wide TB transmission. Furthermore, intense research is required to yield new therapies that limit the acquisition of resistance and reduce treatment duration. Herein, we discuss the possible approaches for addressing these issues, arguing that multifaceted approaches are required, ranging from health systems strengthening for delivering effective diagnosis and patient care to fundamental research on new drug targets.

## Effective health systems for interrupting the transmission of drug resistant TB

Continued transmission will be the key driver to any future burden of TB, with several recent studies pointing to rampant spread of MDR and XDR-TB through deficiencies in the TB care continuum, which include delays in diagnosis, poor linkage to care and lack of retention in the health system (Dheda et al., 2017; Shah et al., 2017; Atre et al., 2022). To address these drivers, health systems require new ways of case finding, faster and cheaper diagnostic tests, together with prophylaxis of contacts to minimise further spread of disease.

### Case finding

Transmission of most infectious diseases is estimated to occur prior to case detection in the health system. Our experience with the management of the recent Covid-19 pandemic has demonstrated that rapid and active case finding approaches were critical to curb further spread of disease (Hossain et al., 2022). Hence, active case finding, rapid diagnosis (including drug resistance profiling) and prompt effective therapy are recognized as the most important interventions to stop transmission (Yuen et al., 2015). Several countries have adopted the FAST (Find cases Actively by cough surveillance and rapid molecular sputum testing, Separate safely, and Treat effectively based on rapid drug susceptibility testing) approach to actively screen for undetected TB and identification of drug resistance, followed by appropriate effective therapy to interrupt transmission. However, for these screening approaches to have a widespread application, cost effective novel methods that do not require sputum but alternative approaches such as active screening through cough surveillance, digital radiology and breath tests need urgent development. Examples of such innovations include the development of machine learning programmes that can successfully distinguish between the cough of a patient with TB or other respiratory illnesses, at a level exceeding the minimum target product profile requirements for a WHO triage test (Pahar et al., 2021). There are already several smartphone applications in development that can analyse coughs and overall coughing trends to detect incidences of respiratory disease (Gabaldon-Figueira et al., 2021; Moschovis et al., 2021). Such approaches need rapid scaling, together with clear processes for accelerating the WHO’s approval and widespread deployment. Recently there has been a renewed focus on non-invasive sampling and using alternate clinical specimens such as oral swabs in diagnosis. For example, the use of cheek swabs have been assessed in adults living with HIV (LaCourse et al., 2022). While tongue swabs were evaluated in young children, a population were traditional sputum samples are not easily obtained (Ealand et al., 2021).

### Case notification

In some countries, TB cases are often not notified, especially those managed in private care where facilities are not linked to national tuberculosis programs (Rupani et al., 2021). As a result, significant under-notification of cases undermines important processes such as disease surveillance and contact tracing. To find more cases, control programs need to expand TB case-detection to populations with increasingly low prevalence of disease. Caution needs to be taken as mathematical modelling demonstrates that poor diagnostic-specificity can result in a high number of false positive diagnoses, giving a skewed picture of program performance leading to inappropriate policy decisions (Lalli et al., 2018). Current diagnostic tests are not suitable for these mass testing approaches. Household contact tracing of index TB cases is promising for TB control but has not been widely implemented, particularly in low-resource settings because of the lack of high quality evidence for effectiveness or due to cost limitations. This would require reallocation of the already insufficient resources available for TB diagnosis and treatment (MacPherson et al., 2019). Dowdy et al. (2017) provided a roadmap for designing, evaluating, and modelling interventions to interrupt transmission in a diverse array of tuberculosis epidemics worldwide. They proposed a three-prong approach for effectively reducing TB transmission that involves (I) preventative therapy to reduce the reservoir of latent infections, (II) diagnosis and case finding to shorten the time from disease onset to initiation of treatment and (III) infection control in certain settings. Although synergistic public health interventions can halt tuberculosis transmission, knowledge and understanding of the local epidemiology is another crucial factor for success (Dowdy et al., 2017).

### Point of care diagnostics

It is imperative that rapid point-of-care (POC) diagnostic tests are developed for early and rapid diagnosis of *M. tuberculosis*. Since 2007, several new tests and diagnostic approaches have been endorsed by the WHO, including: liquid culture with rapid speciation as the reference standard for bacteriological confirmation; molecular line probe assays for rapid diagnosis of multidrug-resistant TB (Hain Lifescience LPA); non-commercial culture and drug-susceptibility testing methods; light-emitting diode fluorescence microscopes; and nucleic acid amplification tests for rapid and simultaneous diagnosis of TB and rifampicin-resistance (Cepheid, GeneXpert; Molbio Diagnostics, TrueNat) (World Health Organization, 2021b). More recently, the Deeplex Myc-TB (Genoscreen) is a culture-free assay based on targeted deep sequencing that is able to provide information on resistance to 15 anti-TB drugs within 48 hours. The Deeplex has 3% sensitivity for the detection of heteroresistance and is also able to identify non-tuberculous mycobacteria, as well as lineages and spoligotypes (Feuerriegel et al., 2021). Despite this progress, an accurate and rapid POC test for drug resistant TB that is usable under field conditions is still unavailable, suggesting that greater research effort is required to convert these often-complex laboratory technologies into robust, accurate and cost-effective POC programs (Abdulgader et al., 2022). The Covid-19 pandemic has expanded the framework for self-testing to enable greater diagnostic pickup of infection, such approaches should be considered and developed for rapid TB diagnosis.

### Whole genome sequencing

The currently available nucleic acid amplification tests detect specific regions of the TB genome to identify probable drug resistance, but new mutations are constantly evolving. Whole genome sequencing (WGS) has the capability to identify all genetic mutations in a sample (Katale et al., 2020), but the use of such technology needs to be refined before it can be implemented in a diagnostic setting. WGS testing would need to be performed directly on sputum in a form that is cheap, fast and accessible but thus far, no such technique that can be deployed at programmatic levels has been reported (Dookie et al., 2022). An additional consideration is that the use of WGS for clinical decision making requires accurate information about genotype to phenotype correlations (Faksri et al., 2019). In the absence of definitive information in this regard, a combination of genotypic and phenotypic approaches will most likely be needed (Dookie et al., 2022). An alternative to WGS is targeted sequencing, which also makes use of next-generation technology to provide information on drug resistance loci directly from clinical samples (MacLean et al., 2020; Dookie et al., 2022). Despite the fact that this technique provides simpler data than WGS, it has the ability to detect low frequency variants (MacLean et al., 2020; Dookie et al., 2022).

### Bacterial culturability

Major limitations of traditional culture methods are slow turn-around times, suboptimal sensitivity, and the prohibitive cost of using automated liquid broth systems in endemic countries. Hence, there is a dire need for improved culture techniques to enhance diagnosis, particularly in certain vulnerable populations. Microbiological confirmation of TB in PLWHIV and in children is challenging due to the paucibacillary nature of disease presentation. Collection of specimens such as induced sputum or gastric aspirate from these groups is complex and invasive often with poor diagnostic confirmation due to limited retrieval of bacteria (Connell et al., 2011). A few novel approaches have been evaluated to improve recovery of *M. tuberculosis* from these limited specimens. These include the colorimetric TK medium assay (Salubris) that changes colour from red to yellow, indicating early positive growth of mycobacteria before visible bacterial colonies appear and also has the capacity for drug susceptibility testing (Kocagoz et al., 2012). The microscopic observation drug susceptibility assay (MODS) which uses an inverted light microscope and Middlebrook 7H9 broth culture containing antimicrobial drugs for susceptibility testing is able to detect early mycobacterial growth as “strings and tangles” of bacterial cells in the media in a shorter time (average of 8 days) compared with Lowenstein–Jensen culture (Moore et al., 2004). Due to limited accessibility of the MODS media, evaluation of two alternative culture media (powder and lyophilized forms) showed equivalent bacterial growth, suggesting that MODS can be implemented in resource limited settings (Sheen et al., 2022). Commercially available bacteriophage-based kits have been developed to detect mycobacteria and identify rifampicin resistance directly in sputum specimens within 2-3 days (Pai et al., 2005). Although TK Medium, MODS and bacteriophage based tests are promising, practical and inexpensive tools, their utility for diagnosis of TB in children and PLWHIV is unknown (Kumar et al., 2015).

The performance and accuracy of culture-based TB diagnostic tests is based on the premise that all bacterial populations have equivalent culturability in routine diagnostic media. However, there is a growing body of evidence that points to bacterial populations adopting a spectrum of physiological states associated with altered culturability. The description of “viable but nonculturable” (VBNC) *Vibrio cholerae* cells more than thirty years ago (Colwell et al., 1985) was just one example of a sub-population of bacterial cells that are incapable of growing on solid medium but can be detected in liquid media. *M. tuberculosis* cells that display the same behaviour are described in the literature, as either differentially culturable tubercle bacteria (DCTB) (Chengalroyen et al., 2016) or differentially detectable (DD) bacteria (Saito et al., 2017). The relative proportion of DCTB in a sample is quantified by comparing the number of culturable bacteria in liquid-limiting dilution assays with that of plateable bacteria on solid media.

Initial descriptions of these cells in mycobacteria were associated with resuscitation promoting factors (Rpfs), originally described in *Micrococcus luteus* as being able to promote the resuscitation of dormant bacteria (Mukamolova et al., 1998). There are five *rpf* genes in the *M. tuberculosis* genome and the associated proteins, which are present in the culture filtrate (CF) of *M. tuberculosis*, have been shown to activate the growth of non-culturable bacteria (Mukamolova et al., 2002; Kana et al., 2008). Both liquid culture dependent and plateable populations of bacteria have been identified in sputum of patients with TB disease, including DCTB populations that can be classed as CF-dependent and CF-independent (Mukamolova et al., 2010; Dhillon et al., 2014; Chengalroyen et al., 2016). Furthermore, the identification of a subpopulation of bacteria able to grow with CF from a *M. tuberculosis* mutant lacking Rpfs suggests involvement of other growth stimulatory factors (Chengalroyen et al., 2016). Individually cAMP and fatty acids appear to offer no benefit in DCTB recovery from sputum samples, contrary to their effectiveness in an *in vitro* model (Shleeva et al., 2013; Gordhan et al., 2021). The presence of these differentially culturable organisms in clinical specimens suggests that approaches to enhance their growth may allow for greater diagnostic pick-up. Indeed, the use of CF and Rpfs allows for the detection of viable *M. tuberculosis* in clinical samples with negative results by standard tests (Dusthackeer et al., 2019). Furthermore, the inclusion of CF is also beneficial for the detection of DCTB in culture negative samples from PLWHIV (McIvor et al., 2021). Recently, *M. tuberculosis* was recovered in a subpopulation of culture negative clinical specimens using lipid-rich media, instead of the standard glycerol-based media (Mesman et al., 2021). DCTB populations in sputum have been shown to be tolerant to a range of anti-TB drugs (Turapov et al., 2016). The number of DCTB in sputum samples increased following the first two weeks of TB treatment, either as a consequence of the antibiotics enriching the number of these bacteria in the samples or by stimulating the generation of new DCTB (McAulay et al., 2018; Zainabadi et al., 2021). However, in patients with drug-resistant TB, the proportion of DCTB did not change significantly following two weeks of treatment, perhaps as a consequence of the administered treatment regimens that excluded rifampicin (Zainabadi et al., 2021).

The presence of co-infections, such as HIV, need to be considered when diagnosing and treating patients with clinical TB. PLWHIV and TB, with CD4 counts >200 cells/ml, are more likely to display higher levels of CF-dependent DCTB then people with CD4 counts <200 cells/ml, suggesting that the host immune system has an effect on the production on these differentially culturable bacteria (Chengalroyen et al., 2016). It has been shown that DCTB are present in the sputum from individuals clinically cured of TB (Beltran et al., 2020). These data underscore that multiple diagnostic tests that account for bacterial phenotypic diversity in sputum are likely required to ensure a true estimation of bacteriological cure rates. This is particularly important when discussing possible alterations to treatment length. Stopping the administration of TB drugs when an individual appears to be cured, and then restarting treatment following relapse of disease, creates the ideal conditions for the emergence of drug tolerant and resistant bacterial populations.

### Addressing heteroresistance

Heteroresistance occurs when only a subpopulation of the bacteria displays the resistance phenotype, or if multiple resistance genotypes occur together. This phenomenon is often unstable, with the resultant higher drug minimal inhibitory concentration (MIC) values being transient and difficult to assess consistently (Andersson et al., 2019). Diagnostic testing of such mixed infections may only identify the one strain, resulting in treatment with inappropriate drugs that would ultimately select for DR strains. There is conflicting data about the extent that heteroresistance plays in treatment outcomes, with some studies showing poor outcomes whilst others show no association (Kargarpour Kamakoli et al., 2017; Shin et al., 2018; Kargarpour Kamakoli et al., 2020; Chen et al., 2021). Development of deep sequencing algorithms to identify minority strain genotypes in mixed infections will be important for early diagnosis of heteroresistance. Whether this facilitates better patient management will require extensive clinical studies which should be prioritized as heteroresistance remains a clear and present threat to the elimination of drug resistant TB.

### Treatment and prophylaxis

Drug-susceptible TB disease can be treated with antibiotic regimens that take 4 to 9 months to complete. A newly proposed 4-month treatment course consists of 2 months of intense rifapentine, moxifloxacin, isoniazid and pyrazinamide therapy, followed by 9 weeks of rifapentine, moxifloxacin and isoniazid (Carr et al., 2022). Alternatively, rifampicin, isoniazid, pyrazinamide and ethambutol can be administered for 2 months, followed by rifampicin and isoniazid for 4 months. The treatment of drug-resistant TB is more complicated and is dictated by the resistance-causing mutations present. The WHO recommended in 2020 that isoniazid-resistant, but rifampicin-susceptible TB, be treated with rifampicin, ethambutol, pyrazinamide and levofloxacin for 6 months. RR-TB and MDR-TB treatment regimens during an intensive 4-month phase consist of levofloxacin/moxifloxacin, clofazimine, ethionamide, ethambutol, isoniazid (high dose), pyrazinamide and bedaquiline (for 6 months). This is followed by a continuation phase with treatment using levofloxacin/moxifloxacin, clofazimine, ethambutol and pyrazinamide for 5 months (World Health Organization, 2020). Individuals infected with TB resistant to any additional antibiotic require individualised treatment regimens that last for 18 months. Potentially new anti-TB compounds currently undergoing clinical trials are listed in **Box 1** (Dartois and Rubin, 2022; Working Group on New TB Drugs, 2022). Given that resistance to bedaquiline, the first drug to be approved for treating TB since the 1960s, is emerging globally, these new drugs are critical for future TB regimens (Chesov et al., 2022; Nair et al., 2022).

A key missing feature for TB control is a comprehensive program for prophylaxis for contacts of index cases with drug resistant TB. The toxicity and high costs of currently available Group B agents (**Box 1**), which would comprise the first choice for prophylaxis, are major stumbling blocks to this. Currently, the National Tuberculosis Controllers Association and CDC recommend short-course treatments (3-4 months) using rifamycin-based regimens for contacts of drug sensitive TB index cases. These could take the form of once-weekly isoniazid and rifapentine for 3 months, 3 months of daily isoniazid and rifampicin or daily rifampicin for 4 months. Six or nine months of daily isoniazid treatment is an available alternative treatment but does come with a higher toxicity risk and the longer course period can result in lower completion rates (Sterling et al., 2020). There are on-going clinical trials looking at preventative measures for household contacts of patients with MDR-TB. PHOENIx (Protecting Households On Exposure to Newly Diagnosed Index Multidrug-Resistant Tuberculosis Patients) is a phase III trial comparing the use of delamanid vs isoniazid for 26 weeks in preventing active TB in high-risk children and adult members of households with active MDR-TB cases (National Institute of Allergy and Infectious Diseases et al., 2021). The TB child multidrug-resistant preventive therapy (TB-CHAMP) phase III trial is addressing the use of levofloxacin to prevent TB disease in children under the age of 5 years, who are contacts of people with active MDR-TB (Seddon et al., 2018). More clinical trials in vulnerable populations such as healthcare workers, PLWHIV and children are needed. Furthermore, the psychosocial complexities of treating people with no (or mild) symptoms with drugs that have side-effects will need to be addressed.

### Transmissibility of drug resistant strains

In the household setting, emerging evidence points to greater risk of transmission with drug resistant TB when compared to drug susceptible disease (Becerra et al., 2019). However, this effect is most likely due to delayed diagnosis or treatment of drug resistant TB rather than an increased transmissibility of resistant strains. Consistent with this a recent study from India confirmed that the high transmission of drug resistant TB, particularly in young people, was associated with delays in care including rapid diagnosis and linkage to treatment (Atre et al., 2022). As such, direct contemporaneous evidence that drug resistant strains are more transmissible in the community is lacking.

### Subclinical TB and transmission

Traditionally, TB transmission has been associated with the individuals who display symptoms and are able to spread bacteria through coughing however, the role of subclinical TB in community transmission has recently garnered interest. Subclinical TB is defined by the presence of tubercle bacilli in the lung with no associated clinical TB symptoms and is radiologically and/or microbiologically detectable (Peters et al., 2019). One mechanism through which individuals with subclinical TB can contribute to transmission is through TB-unrelated chronic cough, possibly associated with air pollution or chronic lung disease (Esmail et al., 2018). Approaches to addressing chronic coughs, including treatment of viral infections may yield potential benefits (Esmail et al., 2018). Recent evidence suggests that tubercle bacteria can also be spread by tidal breathing indicating that various respiratory manoeuvres, including singing and talking, could contribute to TB transmission (Williams et al., 2020; Dinkele et al., 2022). Current estimates suggest that roughly 7-10 million individuals are living with subclinical TB, who most likely have heterogenous clinical trajectories and outcomes which impact on the ability to transmit bacteria (Kendall et al., 2021). These data illustrate that sub-clinical TB could serve as a significant driver of TB transmission. Further studies addressing this will require diagnostic tests that are accurate and easy to scale in large populations, together with appropriate bioinformatics and modelling tools to infer directionality of transmission over long periods of asymptomatic disease, sometimes in complex epidemiological settings (Kendall et al., 2021).

Another aspect to consider when looking at transmission is the mechanistic factors that dictate how the disease moves from one person to another. It has been shown that *M. tuberculosis* produces sulfolipid-1 (SL-1) which is able to activate nociceptive neurons in guinea pigs and trigger coughing (Ruhl et al., 2020). Whether a similar mechanism occurs in humans needs investigation to understand the relationship between the extent of the cough and transmission. The likelihood of transmission could be dependent on the type of *M. tuberculosis* strain present in the lungs. In mice, high transmission strains induce alveolar macrophages, in an interleukin-1 receptor-dependent manner, to migrate into the lung interstitium. This ultimately leads to granuloma formation with potential cavitary lesions, which enable the *M. tuberculosis* bacilli to enter the airways (Lovey et al., 2022). Aggregates of *M. tuberculosis*, when compared to single bacteria, face less acidification in macrophage phagosomes, are associated with an increase in bacterial replication and initiate the death of the macrophage that can lead to a macrophage-death cascade (Mahamed et al., 2017; Rodel et al., 2021). Given that such aggregates have been identified in human granulomatous lesions (Rodel et al., 2021), together with clumps of cells detected in TB patient bioaerosols (Dinkele et al., 2021), suggests that clumping of bacterial cells could be a means of continued transmission and bacterial pathogenesis.

It is clear that extrinsic and environmental factors related to TB diagnosis, treatment and patient behaviour contribute significantly to the emergence of drug resistance in *M. tuberculosis*. Addressing these factors, together with novel health systems strengthening interventions, will significantly assist in curbing the spread of TB. The second aspect to limiting the emergence of drug resistance is developing and understanding of the bacterial factors that drive drug tolerance and the ultimate emergence of resistance. Understanding these factors is crucial to anticipate future trajectories of acquired resistance, identification of potential drug targets and development of more effective shorter regimens. Below, we highlight the contribution of some of these bacterial mechanisms and their possible roles in the development of acquired drug resistance.

## Acquired drug resistance: Bacterial mechanisms for surviving antibiotic treatment

*M. tuberculosis* shares an extremely complex relationship with its human host. These bacteria have adapted to activate specific responses to modulate their physiology and resist stress encountered during pathogenesis. Multiple and complex stress responses induce the development of different bacterial subpopulations, which can display varying levels of tolerance to drugs, resulting in a continuous spectrum of phenotypes that contribute to disease relapse despite protracted treatment regimens. Considering this, current definitions of tolerance and persistence may be too simplistic and require revision to incorporate the associated mechanisms to describe these complex and often coexisting phenotypes.

Upon repetitive and intermittent antibiotic treatment, bacterial populations adapt and eventually become tolerant, manifesting persisters that evade death, even at very high concentrations of drug (Sulaiman and Lam, 2021). Persistent bacteria are characterised by their ability to survive high antibiotic concentrations, without genetic mutations in drug targets/in genes associated drug mechanisms of action. As they are genetically identical to their antibiotic susceptible counterparts, persistence is considered a phenotypic trait used to protect the longevity of the overall population in the face of adversity (Balaban et al., 2019; Huemer et al., 2020; Sulaiman and Lam, 2021). Persistence and the associated drug tolerance can also be defined in the context of clinical infection by lesions that do not clear because of bacterial tolerance or insufficient drug concentrations in lesions as a consequence of poor regimen adherence or inadequate drug diffusion (Sarathy et al., 2016).

### The importance of tolerance as a prelude to resistance

The long-standing debate of whether antibiotic tolerance and resistance are related or two distinct phenotypes, was recently addressed through *in vitro* experiments and mathematical modelling demonstrating that tolerant *Escherichia coli* populations eventually became resistant to drugs (Levin-Reisman et al., 2017). Resistant mutants of *M. tuberculosis* were also shown to arise from antibiotic tolerant persisters explaining the ability of these bacteria to not just survive but replicate in the presence of a drug (Sebastian et al., 2017). Mechanisms contributing to antibiotic tolerance include the activity of efflux pumps, pathway redundancies, direct inactivation of drugs or modifications to the drug target, thus allowing persisters to survive in the presence of antibiotics for long periods of time, with consequent elevated MIC values (Balaban et al., 2019; Sulaiman and Lam, 2021). Combined, these mechanisms that ultimately confer tolerance should be the subject of intense study as they most likely contribute substantively to stable genetic resistance in *M. tuberculosis*. Some of these mechanisms are briefly outlined below.

### Dormancy

Dormant bacteria do not replicate, displaying decreased metabolic activity thereby protecting themselves from antibiotics that require active bacterial growth to be effective. As a result, dormant bacteria display tolerance to many drugs, but through different (and sometimes overlapping) mechanisms to those that confer tolerance in replicating bacteria (Balaban et al., 2019; Tasse et al., 2021). Whilst the phenomenon of non-replicating persistence has been described for mycobacteria *in vitro*, using several different laboratory models (Gold et al., 2015; Gold and Nathan, 2017), there is insufficient evidence that a dormancy phenotype prevails in human TB. It has been reported that mycobacteria are able to sporulate, a metabolic state synonymous with dormancy, but subsequent analyses suggested that this is probably unlikely given the lack of genetic orthologues that mediate the process (Ghosh et al., 2009; Traag et al., 2010). Mycobacteria do retain the capacity to adopt non-replicative states that are associated with morphological changes however, the role of these changes in conferring antibiotic tolerance remains largely unexplored (Shleeva et al., 2011). The molecular mechanisms that have been associated with this trait may be useful to develop new TB drugs and many drug screening programs have opted to use laboratory conditions that best mimic non-replicating persistence.

### Heterogeneity in bacteria and lesions

Studies in animal models and directly in individuals with TB have shown the presence of heterogenous and divergent individual lesions within a single host, arising from differential immune potential of host cells and the varied virulence of TB bacilli (Lenaerts et al., 2015; Dhar et al., 2016). The outcome of individual lesions appears to be driven by the local tissue environment rather than the general host response, suggesting a defining role for these microenvironments in *M. tuberculosis* heterogeneity. Heterogeneity in metabolism of individual bacteria has a direct impact on cell growth rates, metabolic processes, stress responses, and drug susceptibility (Coleman et al., 2014; Lin et al., 2014; Gideon et al., 2015; Prideaux et al., 2015; Marakalala et al., 2016). These differences may not confer fitness advantages to the bacterial population under optimum growth conditions but can be beneficial for survival during stress and antibiotic treatment. Whilst there is growing appreciation of bacterial heterogeneity as an important driver of persistence in TB disease, targeting the mechanisms that drive this phenomenon will be difficult without a comprehensive mechanistic understanding of key mediators of the effect. Developing new tools to study this under physiologically relevant conditions emerges as an important research priority.

Asymmetric cell division in mycobacteria is another contributing factor for establishing a heterogeneous population both in size and elongation rate, with varying drug susceptibility (Aldridge et al., 2012; Kieser and Rubin, 2014; Logsdon and Aldridge, 2018). *M. tuberculosis* cells grown *in vitro* under stress conditions, or isolated from sputum or infected macrophages, display increased heterogeneity in cell-size suggesting that heterogeneous populations can have different sensitivity to drugs resulting in variable treatment outcomes (Vijay et al., 2017). Asymmetry in division also results in disproportionate distribution of oxidized proteins between the progeny, which is associated with a fitness cost as cells with a higher content of oxidized proteins grow slower and struggle to recover after exposure to antibiotics (Vaubourgeix et al., 2015). Single-cell heterogeneity in *M. tuberculosis* has also been linked to LamA, a divisome protein that inhibits growth at nascent new poles. Deletion of *lamA* results in loss of heterogeneity and faster killing by vancomycin and rifampicin (Rego et al., 2017).

Other mechanisms contributing to heterogeneity include growth processes such as cell elongation, DNA replication and chromosome segregation. At any given time point during growth, individual cells in a population are in different phases of elongation and division in relation to each other. Mapping of cell division cycles and chromosomal replication at the single-cell level in *Mycobacterium smegmatis* has identified cell-to-cell heterogeneity in growth rates and interdivision times, together with changes in duration of various cell cycle periods, resulting in large variations in cell size, age, ploidy, and generation times (Santi et al., 2013; Santi and McKinney, 2015). Similarly, mapping of the cell cycle in *M. tuberculosis* would enhance our understanding of bacterial heterogeneity in the context of protracted treatment and/or treatment failure. Growth rate variations dependent on nutrient availability is another widely studied characteristic contributing to heterogeneity due to the strong link between proliferation and virulence, together with the relationship between growth arrest and drug tolerance (Ray et al., 2011; Cerulus et al., 2016; Hashimoto et al., 2016).

### Translesion DNA synthesis and mutagenesis

Maintenance of genomic integrity by dedicated repair systems is vital to prevent blockage of DNA replication associated with replication fork collapse. In addition to a host of DNA repair pathways, mycobacteria also have translesion DNA polymerases that are able to transiently replace the replicative polymerase to bypass lesions resulting from DNA damage. This allows for DNA damage tolerance and mutagenesis (Fuchs and Fujii, 2013). DnaE2, an alternate homologue of the replicative polymerase is associated with UV tolerance and UV-induced mutagenesis, leading to the emergence of drug resistance (Boshoff et al., 2003; Warner et al., 2010). More recently, the overexpression of DinB1, a Y-family DNA polymerase was shown to promote missense mutations in the *rpoB* gene, conferring resistance to rifampicin (Dupuy et al., 2022). The mutational signature for rifampicin resistance displayed by DinB was distinct from that of DnaE2, implicating translesion synthesis/template slippage on homopolymeric runs as a strong driver of mycobacterial genome diversification with antimicrobial resistance and host adaptation implications.

### Compensatory mutations

Drug resistance in *M. tuberculosis* is exclusively due to chromosomal mutations that may confer drug resistance *via* modification or overexpression of the drug target and/or prevention of prodrug activation. These modifications may impart pleiotropic effects leading to a reduction in bacterial fitness (Andersson and Hughes, 2010) which can be mitigated by compensatory mutations by interacting epistatically with the resistance mutation (Comas et al., 2011; Gygli et al., 2017). The clinical relevance of the evolution of compensatory mutations is still poorly understood. When compared to other pathogenic bacteria, the genetic diversity of *M. tuberculosis* is low suggesting that the accumulation of compensatory mutations may have important implications for the stability of drug resistant phenotypes (Ford et al., 2013).

### Transcription

Promoter specificity in *M. tuberculosis* is driven by 13 sigma factors, small proteins that bind to the RNA polymerase holoenzyme in response to specific environmental stimuli (Rodrigue et al., 2006; Paget, 2015). Sigma factor E, SigE, is expressed in response to surface stress, low pH, oxidative stress, several antibiotics and has been proposed as a major mediator for the switch to non-replicating persistence (Balazsi et al., 2008; Dona et al., 2008; Manganelli and Provvedi, 2010; Tiwari et al., 2010; Manganelli, 2014; Pisu et al., 2017). Two SigA dependent promoters upstream of the *rpoB* gene, the target for rifampicin, have been shown to be responsible for semi-heritable tolerance in mycobacteria. Rifampicin preferentially inhibits one of the two promoters, whilst the second promoter is induced in the presence of rifampicin, resulting in accumulation of RpoB in the cytoplasm, thus supporting growth in the face of bactericidal rifampicin concentrations (Zhu et al., 2018). Mycobacteria also utilize a two-step pathway involving the glutamine amidotransferase (GatCAB) enzyme to regulate translational fidelity of glutamine and asparagine codons as an adaptive survival strategy. Mistranslation of the *rpoB* gene has been shown to reduce production of RNA polymerase and increase bacterial survival in the presence of rifampicin (Su et al., 2016). The collective role of the *M. tuberculosis* stress response pathways in drug tolerance and persistence provide an opportunity to target sigma factors as alternative strategies to shorten TB treatment.

### Toxin-antitoxin systems

*M. tuberculosis* encode 88 toxin-antitoxin (TA) modules, that play a role in regulating adaptive responses to stresses generated by the host environment and drug treatment (Slayden et al., 2018). Most TAs are classified as type II, characterized by a toxin with endoribonuclease activity and an antitoxin that binds the toxin to neutralize its activity. Induction of antitoxin degradation is specific to the environmental stimuli, allowing the toxin to exert its effect leading to reduced metabolism and cell division arrest (Barth et al., 2019; Bordes and Genevaux, 2021). *M. tuberculosis* mutants carrying deletions in one or more of these TA systems display greater susceptibility to drugs compared to the parental strain. Different TA systems have been shown to display specific, as well as diverse roles in the presence of drugs (Singh et al., 2010; Tiwari et al., 2015). Given that TA modules directly alter metabolism, future studies assessing the effects on single cells will be valuable to determine how these systems contribute to persistence development in *M. tuberculosis*. Another challenge that needs to be addressed is the redundancy of these systems in *M. tuberculosis*. New drugs would need to target all systems concurrently or target a master metabolic mediator.

### Biofilms

Mycobacterial biofilms *in vitro* are characterized by the formation of a floating pellicle at the air-medium interface. This phenomenon is of particular interest as cells that survive drug treatment generally are enriched in biofilms due to reduced growth and/or metabolism as well as reduced accessibility to drugs. Mycobacterial biofilms show extreme tolerance to isoniazid, and contain elevated numbers of persisters that survive high concentrations of rifampicin (Ojha et al., 2008). A Tn-seq screening of *M. tuberculosis* mutants unable to form biofilms identified genes responsible for biofilm formation that were also involved in stress and drug tolerance, suggesting that the hostile environment of biofilms, characterized by nutrient and oxygen depletion, has the potential to increase the proportion of persisters (Richards et al., 2019). Whilst initial studies suggested the presence of mycolic acids in biofilms, subsequent analyses pointed to the presence of cellulose in the extracellular matrix of mycobacterial biofilms (Trivedi et al., 2016). In fact, structural components of biofilm matrices are dependent on the *in vitro* model used to generate the floating pellicles (Chakraborty and Kumar, 2019). Recently, the relevance of drug tolerant biofilms to human TB was demonstrated in non-human primates and in lung tissue sections obtained from individuals with TB (Chakraborty et al., 2021). *M. tuberculosis* strains defective in biofilm formation were attenuated for survival in mice, suggesting that biofilms protect these bacilli against host immunity assaults. Moreover, mice infected with *M. tuberculosis* and subsequently treated with a cellulase to degrade biofilms displayed increased susceptibility to isoniazid and rifampicin, suggesting a role for biofilms in phenotypic drug tolerance (Chakraborty et al., 2021).

### Metabolism

Many aspects of mycobacterial metabolism contribute to drug tolerance, we will focus here on a select few. Exposure of *M. tuberculosis* to stresses such as low iron, pH, or oxygen, results in the accumulation of large amounts of intracellular triacylglycerol (TAG) droplets. *M. tuberculosis* cultures from growth-limiting conditions accumulate TAG and display increased tolerance to several drugs, an effect dependent on a functional *tgs1* gene (Deb et al., 2009; Baek et al., 2011). TAG accumulation has also been implicated in carbon storage for rapid restoration of metabolic activity during resumption of growth (Daniel et al., 2004), and in the maintenance of redox homeostasis under conditions of low respiration (Leistikow et al., 2010). In addition, the increased content of intracellular lipid inclusions, including TAGs, in caseum bacilli is linked to the development of mycobacterial caseum-induced tolerance to several first and second line drugs (Sarathy et al., 2018). Persistent bacteria have intracellular lipid bodies and monitoring sputum smears from patients on treatment, with Nile-Red for staining lipid bodies, revealed the presence of persister bacilli that did not clear at the rates as those that formed colonies using conventional culture (Kayigire et al., 2015; Sloan et al., 2015). In this context, combining different staining approaches with conventional culture may yield new clinical endpoints that enable rapid triage of new treatments. However, research in this area has been largely focused on drug sensitive TB. Whether such approaches would be useful for drug resistant TB is unclear and future work should focus on this.

Metabolomic analysis of *M. tuberculosis* treated with sublethal concentrations of isoniazid, rifampicin or streptomycin highlighted the induction of a common subset of metabolites linked to the tricarboxylic acid (TCA) cycle, glyoxylate metabolism and amino acid biosynthetic pathways (Nandakumar et al., 2014). Interrogation of the TCA cycle showed activation of the bifunctional isocitrate lyases (ICL) and methylisocitrate lyases under the oxidative stress conditions generated during antibiotic treatment, with increased activity of the glyoxylate shunt and decreased activity of the reductive arm of the TCA cycle (Kohanski et al., 2007). These data pointed to reduction in respiration to counteract the increased reactive oxygen intermediates (ROI) induced by antibiotic treatment. Consistent with this, a mutant deficient in ICL was associated with induction of several ROI responsive genes, implying that ICL has a role in counteracting endogenous oxidative stress (Nandakumar et al., 2014). These observations point to an urgent need to study the fundamental metabolism of drug resistant isolates of *M. tuberculosis* as these mechanisms may differ significantly, thus requiring a different strategy.

### Phase variation

GlpK, an enzyme required for glycerol catabolism *via* the glycolytic pathway is essential for growth of *M. tuberculosis* in glycerol containing media. Interestingly, *glpK* is subjected to phase variation due to frequent and reversible frameshift mutations in its open reading frame, resulting in some clinical strains producing subpopulations of small colonies with a smooth surface phenotype with heritable tolerance to several drugs. Accumulation of these variants during drug treatment with a rapidly reversible genetic mechanism of drug tolerance has important consequences for treatment failure and relapse (Bellerose et al., 2019; Safi et al., 2019). The frameshift mutations associated with this observation are likely driven by previously mentioned translesion synthesis DNA polymerases, with a reduced fidelity for DNA replication. How these polymerases contribute to phase variation requires further investigation.

### Macrophage activation

The role of efflux pumps in intracellular drug tolerance was validated through treatment of infected macrophages with drug efflux inhibitors, which resulted in reduced drug tolerance (Adams et al., 2011). *M. tuberculosis* can grow without any constraint in resting macrophages but replication of the bacilli is severely reduced in activated macrophages where the bacteria encounter a more hostile environment. Single cell studies showed that under nutrient starvation conditions, bacterial phenotypic heterogeneity increased, consisting of non-growing metabolically active and drug tolerant bacterial subpopulations. However, mice lacking interferon-γ did not display this phenotypic heterogeneity, suggesting that the level of macrophage activation may play a role in inducing drug tolerance (Manina et al., 2015). Indeed, sensitivity comparisons of bacteria residing in resting or activated macrophages with the four first line TB drugs showed differential transcriptional responses of *M. tuberculosis* to isoniazid treatment. The genes induced by isoniazid treatment were associated with regulons responsive to low pH, nutrient starvation, nitrosative or oxidative stress, and surface-damage. Exposure of intracellular bacteria to individual frontline TB drugs yielded a common set of gene expression responses, despite different modes of drug action, suggesting that macrophage activation imposes a stronger stress to the intracellular bacteria (Liu et al., 2016). As such, modulating macrophage function could directly affect drug tolerance mechanisms in tubercle bacilli, a premise of host directed therapies (HDTs), which has emerged as a promising area of research. Current drugs for diabetes, for use as HDTs, promote ROS production leading to mycobacterial killing whilst inhibition of cholesterol synthesis decreased lipid accumulation in animal models when treated together with antitubercular drugs, suggesting a potentially beneficial effect in human TB (Parihar et al., 2014; Singhal et al., 2014; Skerry et al., 2014; Turgeon et al., 2016). However, analysis of data from a national medical claim database showed no beneficial effect of these drugs during *M. tuberculosis* infection (Kang et al., 2014) suggesting the need for more controlled clinical trials to help understand the relevance of HDTs in TB infection. It is also important to assess and understand the possible drug-drug interactions between HDT and currently administered TB drug regimens for successful outcomes.

### Immunometabolism

Immuno-metabolism during TB infection has important implications for pathogenesis and disease outcomes, with recent studies pointing to notable differences between drug resistant and drug sensitive TB. Following TB treatment, there are differences between the regulatory T cells of the immune system in patients with either drug susceptible or drug resistant TB, with these changes still being evident in drug resistant patients after 6 months of treatment (Tellez-Navarrete et al., 2021). Rifampicin resistant TB results in the down regulation of 35 tripartite motif (TRIM) proteins in blood leukocytes when compared to drug sensitive TB (Liu et al., 2021). A coinfection of diabetes and TB results in systemic inflammation, leading to poor outcomes (Kumar et al., 2019). Additionally, drug resistant TB strains, including RR- and MDR-TB, have been shown to be associated with an increased risk of diabetes, treatment failure and death (Kang et al., 2013; Ruesen et al., 2020). While infection with TB results in significantly higher levels of nitric oxide (NO) indicators when compared to healthy controls, these levels are not as high when the infection is with MDR-TB. Following two months of treatment, the NO levels in those individuals with drug sensitive TB returned to normal in a manner that was not seen in individuals with MDR-TB, perhaps indicating a lower level of immunological response (Butov et al., 2014). MDR and XDR-TB infected macrophages induce high levels of oxidative stress, which, as described previously, can have direct implications for drug efficacy (Tyagi et al., 2020).

MDR-TB affects various characteristics of host leukocytes, such as prolonging the circulation of a range of monocytes but decreasing the frequency of HLA-II expressing monocytes, resulting in a proinflammatory status with high IFN-γ and TNF levels months after TB treatment (Ocana-Guzman et al., 2021a; Ocana-Guzman et al., 2021b). This effect is not seen with drug susceptible TB. While inflammatory signalling and TCA cycle remodelling occurs during drug susceptible TB infection, 2 months of ineffective treatment of MDR-TB resulted in a marked upregulation of this inflammatory cascade, an effect that took a year to reverse (Collins et al., 2021). These, and related effects, should be carefully investigated to develop appropriate health systems strategies for monitoring outcomes of drug resistant TB.

Although the field of antibiotic persistence and tolerance has made great advances, much still needs to be uncovered to fully understand how these multiple mechanisms interact to generate beneficial phenotypic heterogeneity for the survival of the bacterial population during pathogenesis. Novel therapeutics aiming to eradicate persisters are desperately needed to shorten TB treatment. In addition, novel cutting-edge technologies for detecting persister bacteria in clinical specimens are urgently needed.

## Covid and TB

Globally, the Covid-19 pandemic has set back the fight against TB by a decade, which is estimated to result in an additional 6.3 million people developing TB and an additional 1.4 million deaths between 2020 and 2025 (Dheda et al., 2022). In South Africa, there was a 50% reduction in the number of TB tests conducted during the initial stages of the Covid-19 pandemic. Added to this was the 12% reduction in the targeted TB treatment success rate of 90%. In response, several provinces in South Africa have developed aggressive TB plans to help monitor the TB response (Jeranji, 2021). An example is the launch of a first-of-a kind public facing TB dashboard in the Western Cape Province. The TB dashboard which includes data on TB cases, deaths, test positivity and drug resistance, from 2015 onwards was modelled based on lessons learnt from the global response in the fight against Covid-19. This interactive program aims to get the TB response in the province back on track (Jeranji, 2021). Similarly, researchers in India and across the world have called for real-time TB dashboards for recording cases and deaths (Mascarenhas, 2022).

Another effective intervention for the management of Covid-19 are mobile phone screening/tracing apps (Ruhwald et al., 2021). Based on this success, the Department of Health in South Africa has recently introduced a WhatsApp and SMS-based TB screening app called the TB Health Check app that links individuals to TB testing stations. A 12.8% TB positivity was recorded from 30 000 screens (Dheda et al., 2022). Integration of the Covid-19 and TB screening apps is currently underway with possible expansion to include HIV self-testing apps, which have already shown great promise (DiAndreth et al., 2020; Pai et al., 2021).

The Covid-19 experience has sent out a strong message that developing health interventions such as vaccines in a short space of time is achievable. Development of new tools to control the pandemic was only possible because of adequate funding and political support. TB receives only a fraction of the funding compared to Covid-19, clearly raising important questions regarding the financial disparity between these two infectious diseases. Arguably, Covid-19 may have attracted significantly greater financing compared to TB since the health, social, and economic impacts have affected not only poor countries but also wealthy ones. This was evident by the unequal rollout of Covid-19 vaccines, which prioritized vaccinating populations in wealthy countries over poorer nations (Treatment Action Group, 2021).

## Future perspectives

The continued spread of resistant pathogens will undermine any gains from modern medicine, plunging health care back to the pre-antibiotic era and creating the ideal environment for emergence of new pandemics at a global scale. Health systems need to urgently strengthen those processes that interrupt TB transmission in communities, hospitals and other congregate settings to rapidly eliminate new infections. Developing scalable and affordable genomic surveillance mechanisms for drug resistant TB in all countries will be central to limiting the spread of new infections. In addition, to prevent full blown drug resistant TB, prophylaxis of vulnerable contacts should be an urgent priority, with the outlook to achieving full coverage with reduced side effects. Given the rapid emergence of drug resistance, new research that uncovers vulnerable drug targets and highlights those processes that are fundamental to mediating drug tolerance, persistence and ultimately resistance must become urgent global priorities. Mycobacterial metabolism represents a significant departure from other well-studied bacterial systems and in this context, developing a broad knowledge based on fundamental metabolism that can be used to facilitate new drug development will require long-term investment from funding bodies. Public-Academic-Private partnerships will also serve as strong enablers to progress new compounds through the drug development pipeline. Meaningful gains in TB programs can only be achieved with this coordinated approach.

## Author contributions

DL, BG, and BK collectively conceived the overall concept of the review and wrote the manuscript. All authors contributed to the article and approved the submitted version.

## Funding

This work was funded by the Department of Science and Innovation (grant number DCE015) and the South African Medical Research Council (grant number KABV017).

## Acknowledgments

We would like to thank Dr. Christopher Ealand and Dr. Edith Machowski for critical appraisal of the manuscript.

## Conflict of interest

The authors declare that the research was conducted in the absence of any commercial or financial relationships that could be construed as a potential conflict of interest.

## Publisher’s note

All claims expressed in this article are solely those of the authors and do not necessarily represent those of their affiliated organizations, or those of the publisher, the editors and the reviewers. Any product that may be evaluated in this article, or claim that may be made by its manufacturer, is not guaranteed or endorsed by the publisher.
